# The Impact of Unscaffolded GenAI Use on Pre-Service Teachers’ AI Readiness, Self-Regulated Learning, Critical Thinking, and Instructional Design Performance: A Quasi-Experimental Study

**DOI:** 10.3390/bs16071114

**Published:** 2026-07-03

**Authors:** Jun Zhang, Yuting Peng, Xinyue Deng, Qin Zeng, Kai Wang

**Affiliations:** 1 College of Teacher Educations, Southwest University, Chongqing 400715, China; zhangyi1231@swu.edu.cn (J.Z.); pyt277815@email.swu.edu.cn (Y.P.); dxy19122517618@email.swu.edu.cn (X.D.); zengqin22@email.swu.edu.cn (Q.Z.); 2 Center for Teacher Education Research, Beijing Normal University, Beijing 100875, China

**Keywords:** GenAI, unscaffolded GenAI use, AI readiness, self-regulated learning, critical thinking, instructional design competence

## Abstract

Although GenAI has been increasingly applied in pre-service teacher education, limited evidence is available on how permitted but unscaffolded GenAI use affects pre-service teachers’ learning and professional development in authentic course contexts. Grounded in cognitive load theory and the zone of proximal development, this quasi-experimental study examined the effects of unscaffolded GenAI use in an 11-week instructional design course. Two intact sophomore classes at a normal university participated, with one class permitted to use GenAI without prompt templates or instructional guidance and the other not permitted to use GenAI. Data were analyzed using paired-samples t-tests and a one-way analysis of covariance (ANCOVA). After controlling for pretest scores, no significant group differences were found in AI readiness, self-regulated learning, or critical thinking, whereas the control group showed stronger instructional design performance. Within-group comparisons showed that both groups improved in AI readiness and instructional design performance, but not in self-regulated learning or critical thinking. These findings suggest that, in this course context, unscaffolded GenAI access alone may be insufficient to support pre-service teachers’ professional learning and may be less favorable for their instructional design performance.

## 1. Introduction

Generative artificial intelligence (GenAI) tools, such as ChatGPT and Gemini, have rapidly emerged as transformative technologies in education (Giannakos et al., 2025; Nguyen et al., 2024; Tran et al., 2026). In addition to reshaping how learners access information, plan their learning, and seek feedback (Ammari et al., 2025), these tools are also changing teachers’ professional practices and roles. In teacher preparation, GenAI has shown potential to support the understanding of complex concepts (X. Zhai, 2023) and the development of higher-order thinking in instructional design (G. W. Choi et al., 2024). However, reliance on GenAI without sufficient guidance may reduce teachers’ cognitive engagement in routine tasks (Egara & Mosimege, 2024). Nevertheless, limited evidence is available on pre-service teachers’ learning outcomes and professional development when GenAI use is permitted but not explicitly scaffolded in authentic course contexts.

In teacher education, instructional design practice is an essential context in which pre-service teachers connect pedagogical theories with classroom applications and develop professional competencies (Jordan, 2016). As GenAI tools are increasingly being used to support instructional design practice, engaging pre-service teachers in GenAI-assisted design tasks may shape how they understand, evaluate, and apply these tools in their future professional work (Tran et al., 2026; Yang & Appleget, 2025). Since the value of such experiences may depend on how pre-service teachers use GenAI, instructional design courses provide a meaningful context for examining the educational consequences of permitting GenAI use without explicit guidance or prompt scaffolding.

ZPD theory emphasizes the role of instructional scaffolds in helping learners progress toward more independent performance (Vygotsky, 1978; Wood et al., 1976), while Cognitive Load Theory suggests that effective support should reduce extraneous cognitive load and help learners allocate limited cognitive resources more efficiently (Sweller et al., 1998). However, before designing such scaffolds, it is important to understand how permitting GenAI access without guidance is related to pre-service teachers’ learning and professional development in authentic course contexts. Without such course-based evidence, even well-intentioned scaffolds may become superficial or overly restrictive. Although existing research has predominantly examined the functions and affordances of GenAI for teachers, less attention has been paid to teachers’ strategic and reflective engagement with GenAI in authentic course contexts (Qian, 2025). Accordingly, this study employs a quasi-experimental design to examine differences in pre-service teachers’ instructional design performance, AI readiness, self-regulated learning (SRL), and critical thinking under permitted but unscaffolded GenAI-use conditions.

## 2. Literature Review

### 2.1. Applications of GenAI in Education

The use of artificial intelligence in education has evolved over several decades. Among its various forms, generative artificial intelligence has attracted growing attention due to its strong capacity to produce coherent and human-like text. The release of ChatGPT in late 2022 made advanced AI technologies more accessible to the public and further accelerated their adoption in both formal and informal educational contexts (Han et al., 2025). Students increasingly use GenAI, often under the guidance of teachers or course frameworks, to support writing (McGuire et al., 2024), reading comprehension (Pan et al., 2025), and problem solving (Zhou et al., 2024). For teachers, GenAI can provide support by generating teaching materials, such as lesson plans, quizzes, and examples, and by offering inspiration for instructional design (Kohnke et al., 2025; Ng et al., 2024). Research has also shown that, when used with appropriate guidance, GenAI may help reduce learning anxiety and enhance students’ confidence and self-efficacy (Fuchs, 2023). However, much of this research has been conducted in contexts where GenAI use is explicitly guided, such as when teachers provide prompting frameworks, courses embed guidelines for GenAI use, or platforms offer structured support. These forms of support may reduce learners’ burden of independently evaluating the quality of GenAI outputs and thus constitute an important condition for the positive effects reported in prior studies.

At the same time, the widespread use of GenAI has raised concerns about excessive reliance, hallucinated information, and insufficient critical evaluation, especially when learners lack adequate scaffolding or digital literacy (L. Fan et al., 2025; van den Berg & du Plessis, 2023). Therefore, the effects of GenAI use should not be assumed to be inherently positive, but should be understood in relation to the conditions under which it is used. Nevertheless, relatively little is known about the educational implications of permitted but unscaffolded GenAI access in authentic pre-service teacher education contexts. Examining this issue is necessary for clarifying the educational consequences of permitting GenAI use without explicit instructional support in pre-service teacher education.

### 2.2. Unscaffolded Use of GenAI

Scaffolding theory emphasizes the central role of structured guidance in novices’ professional development (Wood et al., 1976). Although the concept was originally developed to describe expert support in learning, later research has extended it to technology-mediated learning environments. For example, traditional computer-based learning environments have been shown to scaffold students’ learning in well-structured tasks (Azevedo & Hadwin, 2005), and scholars have argued that scaffolding in digital contexts should not be limited to direct expert assistance but can also include cognitive support provided by technological tools (Pea, 2018; Yelland & Masters, 2007). Building on this expanded understanding, GenAI has the potential to function as a cognitive scaffolding tool because of its interactivity and capacity for immediate responses. However, whether GenAI can actually function as a scaffold depends largely on users’ ability to interact with it productively and on whether the learning environment provides appropriate guidance for its use. Therefore, designing appropriate scaffolding for learners’ GenAI use in specific educational contexts has become an important issue in educational research.

The rapid diffusion of GenAI has made pre-service teachers’ use of these tools without scaffolding an increasingly common yet insufficiently examined phenomenon. Drawing on perspectives from self-regulated learning, this study defines unscaffolded GenAI use as learners’ self-initiated and self-regulated engagement with GenAI in the absence of GenAI-specific instructional guidance or pre-designed prompting frameworks (Zimmerman, 2008). Existing research suggests that, under guided conditions where learners receive support for GenAI use, GenAI can serve as both a brainstorming partner for pre-service teachers (Mollick & Mollick, 2023; Tlili et al., 2023) and a source of information (Chan & Hu, 2023), thereby reducing cognitive load and allowing more mental resources to be directed toward higher-order thinking and problem solving (Iqbal et al., 2025). At the same time, however, the use of GenAI may also pose cognitive risks. Without structured GenAI-related coursework or guidance, pre-service teachers in the early stages of their professional development may struggle to meaningfully align GenAI use with learning goals and subject-specific pedagogy (Spasopoulos et al., 2025). They may also be prone to cognitive offloading, whereby excessive reliance on AI-generated content can undermine instructional quality and constrain the development of learners’ critical thinking (Y. Fan et al., 2025; Giannakos et al., 2025). Therefore, examining pre-service teachers’ engagement with permitted GenAI use in contexts where GenAI-specific scaffolding is absent can help reveal the distinctive features of this form of use and provide empirical evidence for designing more targeted guidance and scaffolding.

### 2.3. Pre-Service Teachers’ Instructional Design Performance

The emergence of generative artificial intelligence (GenAI) is transforming instructional design from a largely individual activity into a human–AI collaborative process (Chiu, 2024; Krushinskaia et al., 2024). As a core dimension of teachers’ TPACK-related competence, instructional design is not simply a preparatory process for teaching, but rather an externalized representation of teachers’ professional knowledge structures (Angeli & Valanides, 2009). Existing studies suggest that, when combined with structured supports such as concept mapping, peer assessment, or mind mapping, GenAI can facilitate pre-service teachers’ instructional design by providing design ideas, timely feedback, and cognitive support, while reducing cognitive demands and supporting higher-order thinking (An et al., 2025; Iqbal et al., 2025; C.-C. Liu et al., 2025; Y. Zhang et al., 2025).

It is worth noting that most of these studies were conducted in contexts with explicit course frameworks or teacher guidance, where external support could, to some extent, compensate for pre-service teachers’ still-developing instructional design expertise. In the absence of GenAI-related guidance, learners may lack external criteria for evaluating the pedagogical appropriateness of GenAI-generated outputs, as well as course mechanisms that encourage reflection and iteration. As a result, pre-service teachers may be more likely to accept GenAI-generated content uncritically (Yang & Appleget, 2025). Y. Sun and Huang (2025) indicated that GenAI can play a positive role as a direct information source in the initial stages of collaborative instructional design among pre-service teachers, but its contribution tends to decrease during later iterative stages, and its role as a cognitive scaffold remains limited. To better support deeper cognitive engagement, GenAI needs to be complemented by structured scaffolds or human guidance. Therefore, to determine whether and how GenAI can support the development of pre-service teachers’ instructional design competence, it is necessary to first understand their engagement with permitted GenAI use in formal learning contexts without GenAI-specific guidance.

### 2.4. Pre-Service Teachers’ AI Readiness

Teachers are positioned at the front line of AI use in education, and their readiness for AI is a key condition for the successful adoption of AI-enhanced educational practices. AI readiness refers to teachers’ preparedness to use artificial intelligence in education in terms of cognition, ability, vision, and ethics (X. Wang et al., 2023). Teachers with higher AI readiness are more likely to integrate GenAI into their teaching practices and support their professional effectiveness through adaptive and innovative use (Bhargava et al., 2021; Jöhnk et al., 2021; Luckin et al., 2022). In contrast, lower readiness may lead teachers to perceive AI as a technological threat or avoid its use due to limited confidence (Chounta et al., 2022). Overall, existing research under structured support conditions suggests that GenAI activities and AI literacy training may foster pre-service teachers’ AI readiness by improving their attitudes, awareness, and confidence in AI use (Bilbao-Eraña & Arroyo-Sagasta, 2025; Y. Liu et al., 2025; C. Zhang et al., 2023).

In the absence of GenAI-specific guidance, direct engagement with GenAI may have both positive and negative implications. On the one hand, practical experience with GenAI may reduce technology-related anxiety and enhance confidence in using AI tools, thereby supporting the development of AI readiness among pre-service teachers (Sat, 2025; K. Wang et al., 2024). On the other hand, without ethical guidance and mechanisms for critical reflection, unscaffolded GenAI use may remain superficial and fail to substantially improve pre-service teachers’ attitudes toward AI (Katsantonis & Katsantonis, 2024). It may also lead to inflated or inaccurate self-assessments of competence, as pre-service teachers may attribute successful task completion with GenAI support to their own ability development, even when their underlying AI readiness has not substantially improved (Guan et al., 2025; S. Zhang et al., 2026). Given these mixed possibilities, examining AI readiness in the context of GenAI-assisted instructional design learning may provide preliminary insights for the future design of scaffolding that supports pre-service teachers’ meaningful and responsible use of GenAI.

### 2.5. Pre-Service Teachers’ Self-Regulated Learning Ability

Self-regulated learning (SRL) refers to learners’ active construction of meaning and their capacity to monitor and regulate cognition, motivation, and learning behaviors throughout the learning process (Zimmerman, 2013). SRL is particularly important in contexts where external structural support is limited, as learners are required to independently set goals, manage their learning pace, and dynamically adjust their learning strategies (Azevedo & Cromley, 2004). Emerging research suggests that, under conditions of GenAI-related structured guidance, collaboration with GenAI may support learners’ metacognition and SRL skills (Yan et al., 2024). GenAI tools can support this process by helping learners clarify goals and develop learning plans (Hew et al., 2023), as well as by offering real-time feedback and suggestions during learning (Chen, 2024). In addition, GenAI may foster metacognitive awareness by prompting learners to reflect on their performance and evaluate their learning strategies (J. C.-Y. Sun et al., 2023; Takagi et al., 2023).

However, when GenAI-related guidance is absent, the relationship between GenAI use and SRL appears to be more complex. In a quasi-experimental study, Xu et al. (2025) found that students’ SRL may decline in GenAI-supported environments without metacognitive support, suggesting that while GenAI can assist learners in completing tasks, it may also weaken their self-regulatory efficacy. Similarly, when critical thinking or GenAI-related guidance is limited, learners may adopt AI-generated content less reflectively, increasing over-reliance and reducing independent cognitive engagement (Yeung et al., 2026; C. Zhai et al., 2024). These risks may be more pronounced among learners with weaker SRL-related profiles, who are more likely to show passive dependence and fragmented strategy use (Ma et al., 2025; Weng et al., 2024; Xia, 2025). Taken together, prior studies indicate that GenAI may support SRL when appropriate guidance is provided, but its role under unscaffolded conditions remains less clear. This is particularly relevant for pre-service teachers, who need to regulate their own use of GenAI and evaluate its outputs without GenAI-specific support. Therefore, this study examines how unscaffolded GenAI use is associated with pre-service teachers’ SRL in a formal course context.

### 2.6. Pre-Service Teachers’ Critical Thinking

The development of critical thinking has become a central concern in education in the age of artificial intelligence. Critical thinking is commonly defined as reasonable and reflective thinking directed toward deciding what to believe or what to do (Ennis, 1985). Emerging empirical evidence suggests that unscaffolded GenAI use may create conditions that are unfavorable for critical thinking development. For example, studies have reported that learners who rely on AI tools without protective mechanisms or guidance may show weaker independent task performance, reduced cognitive engagement, or overreliance on AI-generated outputs (Bastani et al., 2025; Kosmyna et al., 2025). For pre-service teachers, such risks are particularly relevant because uncritical acceptance of GenAI-generated content may lead them to overlook its fit with specific student groups, curriculum goals, and pedagogical approaches, thereby producing homogenized instructional designs (Mishra et al., 2023).

However, scaffolding may substantially change this cognitive pattern. A quasi-experimental study showed that, compared with an unscaffolded condition, structured prompting or full scaffolding promoted learners’ critical verification and reflective integration of AI outputs rather than simple content extraction (Melanou & Beege, 2026). In the context of pre-service teachers, Tran et al. (2026) through a process mining study, also found that teachers without training in AI thinking frameworks tended to show a shallow pattern of “linear extraction,” whereas those with reflective scaffolding demonstrated more iterative and critical practices involving review and revision. Therefore, examining critical thinking under permitted but unscaffolded GenAI-use conditions may help clarify whether pre-service teachers critically evaluate and adapt AI-generated content during instructional design, while offering preliminary insights for the future design of more targeted scaffolds for GenAI use.

### 2.7. Research Objectives and Research Questions

Although previous research has suggested that GenAI-supported learning can foster the development of pre-service teachers’ professional competence, most existing studies have predominantly focused on GenAI use under guided conditions, leaving underexplored pre-service teachers’ engagement with permitted GenAI use in authentic course learning and professional development when GenAI-specific scaffolding is absent. To fill this gap, this quasi-experimental study investigates the effectiveness of unscaffolded GenAI use among pre-service teachers in an instructional design course.

The selection of outcome variables was informed by both the learning context and the theoretical considerations of unscaffolded GenAI use. Instructional design performance was included as a primary outcome because the study was situated in an instructional design course and such competence represents a core professional requirement for pre-service teachers’ future practice. SRL and critical thinking were selected because, without GenAI-specific scaffolding, learners may need to monitor their own learning processes and critically evaluate AI-generated outputs more independently. These two capacities are therefore particularly relevant for understanding learning in unscaffolded GenAI-use contexts. AI readiness was further included to examine whether pre-service teachers’ perceptions of and readiness for AI changed after use, which may help clarify where structured support is needed in future scaffold design. Based on the reviewed literature, this study tentatively proposes that unscaffolded GenAI use may be associated with changes in these outcomes, while the direction and magnitude of such changes remain to be empirically examined.

The research questions are as follows:

**RQ1.**  *Compared with traditional learning, does permitted but unscaffolded GenAI access influence pre-service teachers’ instructional design performance, AI readiness, self-regulated learning, and critical thinking?*

**H1.**  *Pre-service teachers with permitted but unscaffolded GenAI access are hypothesized to show differences in instructional design performance, AI readiness, self-regulated learning, and critical thinking compared with those in traditional learning conditions.*

**RQ2.**  *What changes occur in pre-service teachers’ instructional design performance, AI readiness, SRL, and critical thinking from pretest to posttest within the experimental and control groups?*

**H2.**  *Some degree of change may occur in instructional design performance, AI readiness, self-regulated learning, and critical thinking from pretest to posttest within both the experimental and control groups.*

## 3. Materials and Methods

### 3.1. Design

This study aimed to examine the effects of GenAI use without GenAI-specific scaffolding on pre-service chemistry teachers’ instructional design competence, self-regulated learning, AI readiness, and critical thinking. A total of 52 pre-service chemistry teachers participated in an 11-week course entitled Chemistry Instructional Design. All participants attended one 90 min theoretical session each week. The course mainly focused on chemistry pedagogy and instructional design for senior high school chemistry topics.

At the beginning of each session, the instructor provided both groups with instructional design materials related to the weekly topic, which served as common reference resources for completing the course tasks. At the end of each session, the instructor assigned two reflection questions related to the weekly topic, which students were required to complete and submit during class. In addition, in the first and eleventh weeks of the course, students completed and submitted an instructional design plan based on a designated topic during class, which served as the pretest and posttest tasks, respectively.

In terms of the experimental treatment, the experimental group was allowed to use GenAI tools when completing the reflection questions, developing instructional design ideas, and generating lesson plans. However, no GenAI-specific training, prompting frameworks, or systematic guidance on GenAI use was provided during the course. In contrast, the control group was not allowed to use GenAI to complete these tasks and was required to work independently based only on the course content and the instructional materials provided by the instructor. Both groups were taught by the same instructor, and the teaching content, instructional materials, task requirements, completion time, and submission procedures were kept consistent across the two groups.

### 3.2. Participants

This study adopted a quasi-experimental design. The participants were second-year undergraduate students majoring in chemistry-related teacher education at a normal university in Chongqing, China. All participants were enrolled in the course Chemistry Instructional Design. They had completed foundational coursework in chemistry and related science subjects and were being prepared as pre-service chemistry teachers.

A total of 52 pre-service chemistry teachers participated in the study. They came from two intact classes in the same course. One class was assigned as the experimental group (*n* = 26), and the other was assigned as the control group (*n* = 26). Students in the experimental group were allowed to use GenAI to assist with completing reflection questions, developing instructional design ideas, and generating lesson plans, while also referring to the course materials provided by the instructor. These tasks were completed and submitted during class. Students in the control group followed a conventional learning approach and completed the same tasks during class based only on the course materials provided by the instructor, without using GenAI.

All necessary official permissions were obtained before the implementation of the study. All participants provided written informed consent and participated voluntarily.

### 3.3. Procedure

**Figure 1:**
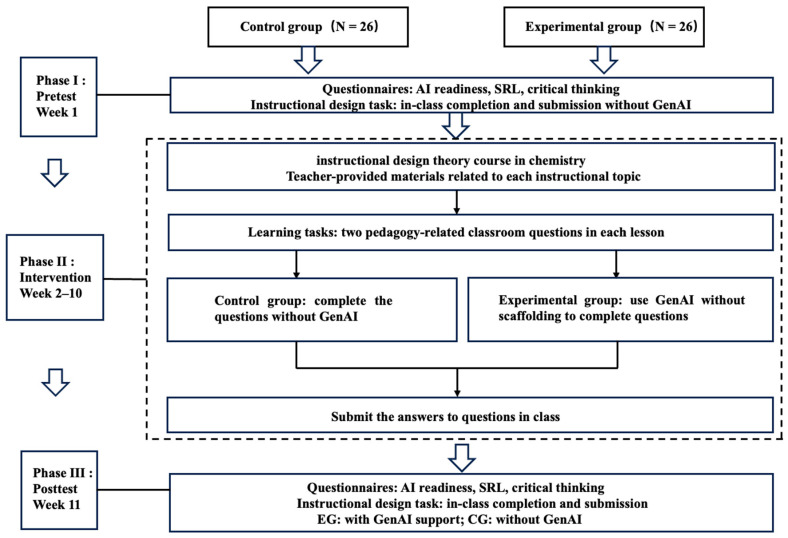
Quasinatural experimental procedure.

This study was implemented in a theoretical course on chemistry instructional design for pre-service teachers. In the experimental group, students were allowed to use GenAI to assist with completing reflection questions, developing instructional design ideas, and generating lesson plans, but no GenAI-specific guidance was provided. In contrast, students in the control group were not allowed to use GenAI for these tasks. The quasi-experimental intervention lasted 11 weeks during the spring semester of the 2024–2025 academic year. The research procedure is shown in Figure 1, and the main steps are described below.

In the first phase, all participants in both the experimental and control groups completed a pretest to assess their initial levels of instructional design performance, self-regulated learning, AI readiness, and critical thinking. The pretest was conducted in the first week of the course. Participants first completed questionnaires measuring self-regulated learning, AI readiness, and critical thinking. They then designed a lesson plan based on the topic and materials provided by the instructor during class. To establish a baseline measure, neither group was allowed to use GenAI when completing the pre-test instructional design task. The completed lesson plans were submitted during class and served as the pre-test products for assessing instructional design performance. In the first week, the instructor mainly introduced the course background and requirements, without providing instruction on specialized course content. All pre-test activities were conducted in class under the supervision of the instructor and a research assistant to ensure consistency in test administration.

During the intervention, the instructor assigned an instructional design topic in each session and provided relevant materials for students’ reference. At the end of each session, the instructor assigned two reflection questions related to the weekly topic. Students in the experimental group were allowed to use GenAI, in addition to the materials provided by the instructor, to complete the reflection questions. They submitted their final responses during class. Students in the control group completed the same reflection questions based on the materials provided by the instructor and submitted their final responses during class without using GenAI.

In the posttest phase, conducted in the eleventh week, all participants completed the same questionnaire measures as those used in the pretest to assess self-regulated learning, AI readiness, and critical thinking. For the instructional design task, participants completed the task under their assigned experimental conditions. Specifically, students in the experimental group were allowed to use GenAI, together with the materials provided by the instructor, to complete and submit a lesson plan related to the topic of that session. Students in the control group completed and submitted the same instructional design task based only on the materials provided by the instructor, without using GenAI. The completed lesson plans were submitted during class and served as the posttest products for assessing instructional design performance.

### 3.4. Instruments

The survey used in this study consisted of two parts. The first part was designed to assess pre-service teachers’ instructional design competence, and the second part was intended to measure their AI readiness, self-regulated learning, and critical thinking. 

The measurement instruments were selected based on their alignment with the main constructs of the study, their use or adaptation in previous educational research, and their reported reliability. Instructional design performance was assessed through students’ lesson design products, as such performance can be more directly captured through authentic instructional design tasks than through self-reported measures. The AI readiness scale was adopted because its four dimensions—cognition, ability, vision, and ethics—were consistent with the purpose of examining pre-service teachers’ readiness to understand, use, and evaluate AI in educational contexts. The SRL scale was selected because self-regulated learning is particularly relevant in contexts where external guidance is limited, as learners need to manage, monitor, and adjust their own learning processes. The critical thinking scale was used because it captures students’ reflective and evaluative thinking, which is closely related to their ability to critically engage with GenAI-generated outputs. These instruments have been used or adapted in previous studies and showed acceptable to high internal consistency. Therefore, they were considered suitable for measuring the relevant constructs in the present study. To minimize potential bias caused by differences in language comprehension, all questionnaires were administered in Chinese.

Students’ instructional design plans from the pre-test and post-test were independently rated by two evaluators using a predefined instructional design assessment rubric. One evaluator was the course instructor, and the other was a high school chemistry teacher. Both evaluators had more than ten years of experience in teaching high school chemistry. Although the evaluators rated the submissions by class, they were not informed whether each instructional design plan was from the pre-test or post-test. The final score for each participant was calculated by averaging the two raters’ scores. To evaluate the reliability of these averaged scores, inter-rater reliability was assessed using the average-measures intraclass correlation coefficient based on a two-way random-effects model with absolute agreement. The results indicated moderate agreement between the two raters for the pre-test scores (ICC = 0.619, 95% CI [0.127, 0.814], F(51, 51) = 3.571, *p* < 0.001, N = 52) and good agreement for the post-test scores (ICC = 0.791, 95% CI [0.635, 0.880], F(51, 51) = 4.724, *p* < 0.001, N = 52). The rubric included seven dimensions: objective design, content analysis, learner analysis, instructional process design, extension design, document quality, and design innovation.

AI readiness was measured using the scale adapted by X. Wang et al. (2023), which included 18 items across four dimensions: cognition, ability, vision, and ethics. Responses were recorded on a five-point Likert scale ranging from 1 (strongly disagree) to 5 (strongly agree). In the present study, the four dimensions were operationalized through items reflecting pre-service teachers’ understanding of teachers’ roles in the AI era, their capacity to integrate AI technologies into classroom routines, their awareness of the strengths and limitations of AI technologies, and their understanding of digital ethics in AI-supported education. Example items were “I clearly understand the new role of teachers in the era of AI,” “I can effectively integrate AI technologies into my classroom routines,” “I understand the strengths and limitations of AI technologies,” and “I understand the digital ethics that teachers should possess in the era of AI,” respectively. The reliability and validity of the scale have been confirmed in previous research, with Cronbach’s alpha coefficients for the four dimensions ranging from 0.90 to 0.97.

Self-regulated learning (SRL) was measured using seven items selected from the SRL scale developed by Lee and Tsai (2011). The instrument comprises seven statements, such as “set my own learning goals” and “explore what I want to learn further”. A five-point Likert scale was used, ranging from 1 (strongly disagree) to 5 (strongly agree). This scale showed good reliability, with a Cronbach’s coefficient of 0.90.

Critical thinking was measured using the scale adapted by Lin et al. (2019). The instrument consisted of six items rated on a five-point Likert scale, with responses ranging from 1 (strongly disagree) to 5 (strongly agree). Example items were “In the process of learning, I will think about whether what I have learned is correct” and “I will try to understand what I have learned from different perspectives”. The Cronbach’s alpha coefficient for this scale in this study was 0.802.

### 3.5. Data Analysis

The study aimed to analyze the effects of unscaffolded GenAI use on pre-service teachers’ instructional design performance, self-regulated learning, critical thinking, and AI readiness in an instructional design course. To achieve this, data analysis was conducted using multiple procedures. First, independent samples t-tests were performed to verify the homogeneity between the experimental and control groups by comparing their pre-test scores. This analysis was conducted to ensure that no significant differences existed between the two groups before the intervention.

Next, to examine the effects of unscaffolded GenAI use (RQ1), one-way analyses of covariance (ANCOVA) were conducted for all outcome variables, including instructional design performance, self-regulated learning, critical thinking, and AI readiness. Additionally, paired samples t-tests were performed within each group to examine changes from pre-test to post-test (RQ2). This analysis allowed for a quantitative comparison of the changes in instructional design performance, self-regulated learning, critical thinking, and AI readiness before and after the intervention in both the experimental and control groups.

## 4. Results

Overall, the results suggest that unscaffolded GenAI use did not produce clear positive effects on pre-service teachers’ SRL, AI readiness, critical thinking, or instructional design performance compared with traditional learning. Although a significant group difference emerged in instructional design performance, the control group outperformed the experimental group. Meanwhile, both groups showed significant pretest–posttest gains in instructional design competence and AI readiness, but not in SRL or critical thinking.

### 4.1. Pretest Results on Pre-Service Teachers’ Instructional Design Performance, AI Readiness, SRL, and Critical Thinking

Table 1 presents the pre-test results for instructional design performance, AI readiness, SRL, and critical thinking. The comparison between the experimental group (EG) and the control group (CG) showed no statistically significant difference, indicating that both groups were equivalent before the intervention.

### 4.2. Posttest Results on Pre-Service Teachers’ Instructional Design Performance, AI Readiness, SRL, and Critical Thinking

#### 4.2.1. Instructional Design Performance

To control for the influence of students’ prior knowledge, a one-way analysis of covariance (ANCOVA) was conducted on the instructional design performance scores, with the pre-test scores as the covariate and the posttest scores as the dependent variable. To determine the suitability of ANCOVA, preliminary checks were performed. Levene’s test revealed that the assumption of homogeneity of variances was not violated (F = 0.005, *p* > 0.05), indicating that the null hypothesis holds and the variances are equal between the groups. Additionally, the assumption of homogeneity of regression slopes was tested, and the results showed that this assumption was also not violated (F = 0.005, *p* = 0.443). The Shapiro–Wilk test of the unstandardized residuals was non-significant, W = 0.977, df = 52, *p* = 0.391, indicating that the residuals did not significantly deviate from normality. Then ANCOVA could be carried out.

In terms of instructional design competence, the average of the instructional design performance scores for the experimental group was 81.50, with a standard deviation of 6.11, while the average for the control group was 86.90 with a standard deviation of 6.67. The adjusted means and standard error of the scores were 81.87 and 1.14 for the experimental group, and 86.54 and 1.14 for the control group. The ANCOVA results indicated a significant between-group difference in posttest instructional design performance after controlling for pretest scores, F = 8.348, *p* = 0.006, $\eta^{2}$ = 0.146. According to the guidelines proposed by Cohen (1988), this effect size can be interpreted as large. Specifically, the adjusted posttest scores of the control group were higher than those of the experimental group under the assigned learning conditions, as shown in Table 2.

#### 4.2.2. AI Readiness

To control for baseline differences between the two groups, a one-way analysis of covariance (ANCOVA) was conducted for AI readiness, with the pre-test score as the covariate and the posttest score as the dependent variable. Preliminary assumption checks were performed before the ANCOVA. Levene’s test indicated that the assumption of homogeneity of variances was not violated, F = 0.000, *p* > 0.05. The test of homogeneity of regression slopes also showed that this assumption was met, F = 0.374, *p* = 0.544. In addition, the Shapiro–Wilk test of the unstandardized residuals was non-significant, W = 0.969, df = 52, *p* = 0.186, indicating that the residuals did not significantly deviate from normality. These results indicated that the data were suitable for ANCOVA.

In terms of AI readiness, the average AI readiness score for the experimental group was 4.07, with a standard deviation of 0.37, while the average score for the control group was 4.11, with a standard deviation of 0.37. The adjusted means and standard errors were 4.07 and 0.07 for the experimental group, and 4.11 and 0.07 for the control group. The ANCOVA analysis showed no significant difference between the experimental and control groups, F = 0.198, *p* = 0.658, $\eta^{2}$ = 0.004, with a negligible effect size. This result suggests that, under the assigned learning conditions, the experimental group did not show a significant advantage over the control group in pre-service teachers’ AI readiness, as shown in Table 3.

#### 4.2.3. Self-Regulated Learning

For SRL, a one-way ANCOVA was conducted using the pre-test score as the covariate and the posttest score as the dependent variable. Preliminary checks indicated that the assumptions of homogeneity of variances and homogeneity of regression slopes were met. Specifically, Levene’s test showed that the assumption of homogeneity of variances was not violated, F = 0.000, *p* > 0.05, and homogeneity of regression slopes, F = 0.003, *p* = 0.955. However, the Shapiro–Wilk test of the unstandardized residuals was significant, W = 0.908, df = 52, *p* < 0.001, indicating that the residuals significantly deviated from normality. This deviation may be partly related to the relatively limited variability in SRL scores. Therefore, the ANCOVA result for SRL was retained but interpreted with caution.

In terms of self-regulated learning, the average self-regulated learning score for the experimental group was 4.15, with a standard deviation of 0.42, while the average score for the control group was 4.15, with a standard deviation of 0.43. The adjusted means and standard errors were 4.15 and 0.08 for the experimental group, and 4.15 and 0.08 for the control group. The ANCOVA analysis showed no significant difference between the experimental and control groups, F = 0.001, *p* = 0.977, $\eta^{2}$ = 0.000, with a negligible effect size. This result suggests that, after controlling for pretest scores, the adjusted posttest self-regulated learning scores did not differ significantly between the two groups under the assigned learning conditions, as shown in Table 4.

#### 4.2.4. Critical Thinking

For critical thinking, a one-way ANCOVA was conducted using the pre-test score as the covariate and the posttest score as the dependent variable. Preliminary checks indicated that the assumptions required for ANCOVA were met, including homogeneity of variances, F = 0.000, *p* > 0.05, and homogeneity of regression slopes, F = 0.029, *p* = 0.864. In addition, the Shapiro–Wilk test of the unstandardized residuals was non-significant, W = 0.967, df = 52, *p* = 0.153, indicating that the residuals did not significantly deviate from normality. In terms of critical thinking, the average critical thinking score for the experimental group was 4.04, with a standard deviation of 0.43, while the average score for the control group was 4.05, with a standard deviation of 0.42. The adjusted means and standard errors were 4.05 and 0.08 for the experimental group, and 4.05 and 0.08 for the control group. The ANCOVA analysis showed no significant difference between the experimental and control groups, F = 0.000, *p* = 0.997, $\eta^{2}$ = 0.000, with a negligible effect size. This result suggests that, after controlling for pretest scores, the adjusted posttest critical thinking scores did not differ significantly between the two groups under the assigned learning conditions, as shown in Table 5.

### 4.3. Changes in the Experimental Group

As shown in Table 6, the paired-samples t-test results for the experimental group showed significant pretest–posttest increases in instructional design performance and AI readiness. Instructional design performance increased from 61.769 (SD = 9.139) in the pretest to 81.500 (SD = 6.114) in the posttest, t = −11.443, *p* < 0.001. AI readiness also increased significantly from 3.748 (SD = 0.368) to 4.066 (SD = 0.373), t = −3.274, *p* = 0.003. However, no statistically significant pretest–posttest changes were found in self-regulated learning or critical thinking. Although self-regulated learning increased slightly from 4.066 (SD = 0.469) to 4.154 (SD = 0.421), the change was not significant, t = −0.736, *p* = 0.468. Similarly, critical thinking increased slightly from 3.968 (SD = 0.427) to 4.038 (SD = 0.428), but this change was also not statistically significant, t = −0.640, *p* = 0.528.

### 4.4. Changes in the Control Group 

As shown in Table 7, the paired-samples t-test results for the control group indicated significant pretest–posttest increases in instructional design performance and AI readiness. Specifically, instructional design performance increased from 64.000 (SD = 8.319) in the pretest to 86.904 (SD = 6.674) in the posttest, and this increase was statistically significant, t = −15.415, *p* < 0.001. AI readiness also increased significantly from 3.806 (SD = 0.409) to 4.114 (SD = 0.372), t = −2.769, *p* = 0.010. However, no statistically significant pretest–posttest changes were found in self-regulated learning or critical thinking. Although self-regulated learning increased slightly from 4.044 (SD = 0.387) to 4.148 (SD = 0.433), the change was not significant, t = −0.978, *p* = 0.338. Critical thinking decreased slightly from 4.090 (SD = 0.472) to 4.051 (SD = 0.424), but this difference was also not statistically significant, t = 0.326, *p* = 0.747.

## 5. Discussion

### 5.1. Effects of Unscaffolded GenAI Use on Pre-Service Teachers’ AI Readiness 

Regarding AI readiness, the results showed that pre-service teachers in the experimental group did not demonstrate significantly higher AI readiness than those in the control group at the end of the course. However, it is worth noting that both groups showed significant improvements in AI readiness over the course.

First, the difficulty of controlling pre-service teachers’ out-of-class GenAI use may provide a potential contextual explanation for this finding. In this study, the classroom task submission procedure was used to ensure, as far as possible, that pre-service teachers in the experimental group used GenAI to complete reflection questions, generate instructional design ideas, and develop lesson plans during class, whereas pre-service teachers in the control group completed the corresponding tasks through traditional learning approaches. However, pre-service teachers’ learning activities after class could not be fully controlled. Over the eleven-week course period, participants in both groups may have encountered GenAI through other courses, informal learning activities, peer communication, or daily digital environments. In addition, both groups’ AI readiness for teaching may have been shaped by the broader visibility of GenAI and the increasing accessibility and ease of use of GenAI tools. Recent research on pre-services teachers’ GenAI adoption also suggests that social influence and tech-savviness are associated with teachers’ behavioral intention to use GenAI (Hao et al., 2026). In this sense, even though GenAI use was not permitted for the control group during classroom tasks, the wider social climate surrounding GenAI and students’ own familiarity with digital tools may have shaped dimensions closely related to AI readiness. This may help explain why AI readiness increased in both groups, while no significant between-group difference was observed at the end of the course.

Second, the nature of the course tasks may also help explain the improvement in AI readiness within the experimental group. Completing instructional design tasks with GenAI within a limited time may have made its perceived efficiency more salient to pre-service teachers, particularly in generating instructional design ideas and draft lesson plans. This may be one possible explanation for the observed increase in AI readiness. Nevertheless, this improvement should be interpreted cautiously because AI readiness was measured through self-reported questionnaires. The increase may partly reflect greater familiarity with GenAI or enhanced perceived competence, rather than fully developed and well-calibrated competence in using GenAI for chemistry instructional design. This interpretation is consistent with Fernandes et al. (2026), who found that although LLM use improved performance on reasoning tasks, users tended to overestimate their own performance. Therefore, whether such improvements reflect substantive development in cognition, ability, and related dimensions of AI readiness requires further examination through scaffolded use and more appropriate forms of assessment.

### 5.2. Effects of Unscaffolded GenAI Use on Pre-Service Teachers’ Instructional Design Performance

Regarding instructional design performance, the exploratory findings of this study showed that when GenAI was used to support instructional design tasks, the experimental group scored lower than the control group. This result suggests that, under conditions where no GenAI-related scaffolding was provided, allowing pre-service teachers to use GenAI did not necessarily support their instructional design performance and may have been associated with lower-quality instructional design outcomes. Two possible explanations may help interpret this finding.

First, unscaffolded GenAI use may have been associated with a misallocation of cognitive resources during the instructional design process. The participants in this study had a strong background in the natural sciences. Shaped by paradigm-based training, they may have been more inclined to seek general principles and more accustomed to rigorous logic, formulas, and quantitative calculation (Zembylas, 2002). In contrast, chemistry instructional design requires pre-service teachers to integrate logical thinking with narrative thinking, that is, to construct and present complex chemistry teaching situations and instructional processes through extended professional writing. For science-oriented pre-service teachers encountering such a highly narrative task for the first time, this shift in thinking may have increased the cognitive challenge of the task.

In this context, the use of GenAI may not have directly translated into higher-quality instructional design. As novice instructional designers, pre-service teachers have relatively limited cognitive resources. For the control group, because GenAI was not involved and the external resources available to them were relatively limited, they may have been more likely to focus their attention on the instructional design theories, subject-specific pedagogical requirements, and lesson-plan writing conventions taught by the course instructor. In contrast, when the experimental group used GenAI to complete instructional design tasks, some of their cognitive resources may have been directed toward repeated interaction with GenAI, prompt revision, and adjustment of generated content. Previous studies have noted that developing effective prompts is itself a skill, and users often need to revise their prompts multiple times before obtaining satisfactory responses (Ammari et al., 2025; Cao et al., 2025; Zamfirescu-Pereira et al., 2023).

When users can clearly identify task needs and adopt appropriate strategies, GenAI may better support instructional design; however, when their purposes are vague or their methods are inappropriate, its value may not be fully realized and may even lead to undesirable outcomes (Wu et al., 2025). This suggests that, in unscaffolded contexts, pre-service teachers may need support not only in how to operate GenAI, but also in clarifying why and when it should be used during instructional design.

Second, the limitations of GenAI in dealing with specialized issues in chemistry education may also have affected the quality of students’ instructional design products. GenAI-generated chemistry lesson plans or instructional outlines may not always sufficiently reflect the disciplinary understanding, instructional logic, and assessment criteria emphasized by the course instructor (Akaygun & Kilic, 2025). Although users can refine these outputs through continued interaction with the system, the improvement gained from this process may sometimes be limited (Emenike & Emenike, 2023). Thus, the lower instructional design scores of the experimental group should not be interpreted as evidence that GenAI necessarily weakens pre-service teachers’ instructional design competence. A more cautious interpretation is that, in a course context without GenAI-related scaffolding, students’ autonomous use of GenAI for instructional design tasks may not have been sufficiently aligned with the disciplinary pedagogical requirements and instructor assessment standards embedded in the course.

Taken together, the experimental results do not necessarily imply that GenAI weakened pre-service teachers’ instructional design competence. Instead, they may suggest that, without appropriate scaffolding, GenAI use may have redirected part of students’ limited cognitive resources from course-based instructional design principles to human-AI interaction and output revision, which may have contributed to a mismatch between their final products and the instructor’s assessment standards.

### 5.3. Effects of Unscaffolded GenAI Use on Pre-Service Teachers’ Self-Regulated Learning 

The exploratory analysis in this study suggests that, compared with traditional learning, unscaffolded GenAI use did not significantly improve pre-service teachers’ self-regulated learning (SRL) when they used GenAI to complete classroom reflection questions, develop instructional design ideas, and generate lesson plans. After the 11-week course, no significant improvement in SRL was observed in either the experimental group or the control group. This finding indicates that simply allowing pre-service teachers to use GenAI autonomously in course tasks may not be sufficient to promote the development of SRL.

One possible explanation is that, in the absence of explicit GenAI-related scaffolding, students’ use of GenAI may have been shaped to a considerable extent by their existing SRL capacity. Students’ SRL ability is widely regarded as a key factor in academic success within digital learning environments (Li et al., 2024). When the course did not provide specific guidance on how to use GenAI for learning planning, process monitoring, outcome evaluation, or strategy adjustment, students may have relied mainly on their own self-regulatory abilities to complete learning tasks (Ma et al., 2025; Xia, 2025). From this perspective, students with relatively high SRL may have been able to manage their learning effectively regardless of whether GenAI was available, whereas students with relatively low SRL may have found it difficult to activate or develop relevant strategies without external support. This may partly explain why pre-service teachers’ SRL did not show significant change after the course. 

Another possible explanation concerns how students positioned the role of GenAI during learning tasks. In the present study, although the experimental group was allowed to use GenAI, the course did not provide any GenAI-related guidance, nor did it explicitly explain how GenAI could support learning goal setting, learning process monitoring, output evaluation, or strategy adjustment. Under these conditions, students may have tended to view GenAI as a convenient source of information or a tool for task completion, rather than as a learning support resource for reflection, monitoring, and strategy adjustment. Previous study have also suggested that, in the absence of structured guidance, learners may not fully use the potential of GenAI to support metacognition and learning strategies (Guan et al., 2025). Therefore, the non-significant change in SRL observed in this study may reflect the limited role of unscaffolded GenAI access alone in promoting pre-service teachers’ SRL. AI Policy Clarity may help students judge what constitutes appropriate GenAI use (Y. Wang et al., 2026). This further highlights the need for tailored scaffolds to guide students’ GenAI use styles and strategies.

### 5.4. Effects of Unscaffolded GenAI Use on Pre-Service Teachers’ Critical Thinking

The results showed that, compared with traditional learning, using GenAI without scaffolding to complete in-class reflection tasks, generate ideas for instructional design, and draft lesson plans was not associated with a significant improvement in pre-service teachers’ critical thinking. In addition, after the eleven-week course, neither the experimental group nor the control group showed a significant pretest–posttest improvement in critical thinking.

One possible explanation is related to the intensity, task design, and implementation of the intervention. Previous research has suggested that even classroom interventions explicitly designed to promote students’ critical thinking may fail to produce significant gains when the intervention intensity or implementation approach is limited (Hou et al., 2026). In the present study, although the intervention lasted for eleven weeks, all classroom tasks had to be completed and submitted during class time. Students were required to complete cognitively demanding tasks within a relatively limited period, either by using the resources provided by the instructor or by drawing on GenAI support. Given the limited cognitive resources available to learners, pre-service teachers, especially as novices in instructional design, may have allocated more attention to understanding task requirements, gathering relevant information, and producing a submittable outcome, rather than carefully examining the accuracy, bias, and pedagogical appropriateness of the information they received (Mayer, 2002). 

Another possible explanation is that critical thinking itself requires learners to judge, analyze, evaluate, and reflect on information, which may further increase the cognitive load involved in learning tasks (Yuxian, 2025). Although critical thinking may support deeper procedural learning, its development often requires sufficient time, clear evaluation criteria, and sustained opportunities for reflection. In the absence of GenAI-related scaffolding, students may be able to obtain information and generate content more efficiently with the help of GenAI, but they may not necessarily be able to identify potential errors, biases, or mismatches between AI-generated outputs and specific teaching contexts. Therefore, unscaffolded GenAI use should not be assumed to translate into the development of critical thinking, particularly when learners have limited experience in instructional design and limited capacity to evaluate AI-generated content.

A further explanation may be related to the measurement method. This study used a self-report questionnaire to assess pre-service teachers’ critical thinking. Although self-report measures can capture students’ perceived critical thinking dispositions or abilities, they may be less sensitive to the judgment, questioning, and evaluation behaviors that students actually demonstrate in specific task contexts. Recent research has suggested that critical thinking in GenAI use may involve more situated and AI-specific processes, such as judging the accuracy of AI-generated information, identifying possible biases or limitations in AI outputs, and considering whether the generated content is pedagogically appropriate for a particular teaching context. These processes may be difficult to capture through a general self-report measure of critical thinking (Lau et al., 2026). Students may also believe that they are already engaging in critical thinking during the learning process, making it difficult for group differences or pretest–posttest changes to be fully captured through self-report measures. Taken together, the non-significant findings in both the between-group comparison and the within-group pretest–posttest comparisons may reflect the combined influence of limited cognitive engagement under unscaffolded GenAI use, the complexity of critical thinking development, and the limitations of self-report measurement.

## 6. Conclusions and Implications

This study examined the effects of pre-service teachers’ use of GenAI for instructional design, in the absence of scaffolded guidance, on their instructional design performance, AI readiness, self-regulated learning (SRL), and critical thinking. The ANCOVA results showed that, after controlling for pretest scores, unscaffolded GenAI use did not lead to significantly higher posttest scores in AI readiness, SRL, or critical thinking compared with traditional learning. Notably, the control group showed significantly better instructional design performance than the experimental group, suggesting that the use of GenAI without guidance may not provide additional benefits for pre-service teachers’ instructional design competence and may even be associated with less favorable performance under certain course conditions. The within-group analyses further showed that both the experimental and control groups improved significantly in instructional design performance and AI readiness from pretest to posttest, whereas no significant improvement was found in SRL or critical thinking.

These findings have several implications for the professional development of pre-service chemistry teachers supported by GenAI, and they also offer tentative practical guidance for teacher educators and the design of teacher education curricula. First, to enhance pre-service teachers’ awareness of GenAI, their perceived ability to use it independently, and their vision for integrating it into future education, it may be beneficial to incorporate GenAI into their everyday learning experiences. Second, the present findings may indicate the limitations of unscaffolded exposure in supporting the development of ethical literacy related to GenAI use. More specifically, the exploratory findings appear to suggest that GenAI use in unscaffolded contexts may have limited potential for promoting pre-service teachers’ SRL and critical thinking. Considering pre-service teachers’ potentially limited pedagogical content knowledge and cognitive resources, teacher educators may need to carefully consider the cognitive demands of instructional design tasks, understand the current status of pre-service teachers’ professional development, and break down complex instructional design tasks into manageable parts. Research has shown that guiding learners through the problem-solving process by providing clear step-by-step solutions can effectively reduce extraneous cognitive load (W. C. Choi et al., 2024; Xinogalos et al., 2017), and this principle may extend to GenAI-integrated instructional design contexts as well. At different stages of instructional design, it may be valuable to clarify learning goals and deliberately guide pre-service teachers to use GenAI according to the purposes of specific tasks, so that they can recognize both its strengths and limitations under different task conditions and define its role appropriately at each stage. In this way, they may gradually develop the ability to use GenAI appropriately. GenAI can serve multiple roles, such as tutor, learner, or learning partner. Exploring its role positioning across different application scenarios and examining potentially effective ways to use it therefore represents a promising direction for future research and practice (Hwang & Chen, 2023).

## 7. Limitations and Future Directions

Although the present study provides some useful insights, several limitations should be acknowledged. 

First, the assignment of participants to the experimental and control groups was not fully randomized but was based on existing intact classes. Because students had already been assigned to fixed teaching classes before the study began, random reassignment was not feasible. This quasi-experimental design may have introduced pre-existing differences between the two groups. Although ANCOVA was used to control for baseline differences, it may reduce but cannot fully eliminate the potential influence of such differences. Therefore, the findings should be interpreted with this limitation in mind.

Second, the sample was drawn from two classes at one university in China. The participating pre-service teachers were relatively similar in terms of academic background, course experience, and cultural context, which may limit the generalizability of the findings. In addition, participants’ demographic information, particularly age and gender, was not fully recorded. This limited the extent to which the sample could be fully characterized and may further constrain the interpretation and generalizability of the findings. Moreover, the relatively small sample size suggests that the results should be interpreted cautiously and viewed as exploratory rather than conclusive. It should also be noted that the ANCOVA result for SRL should be interpreted cautiously, as the residuals for this outcome did not fully meet the normality assumption. Future studies should include larger and more diverse samples of pre-service teachers from different institutional, cultural, disciplinary, and professional development contexts in order to obtain more robust and generalizable evidence.

Third, this study mainly relied on self-report questionnaires and instructional design assignments to assess participants’ psychological variables and learning outcomes. Although these measures provided useful quantitative evidence, questionnaire-based measures may not be sufficiently sensitive to capture subtle changes in learners’ cognition, motivation, and behavior during the learning process. In addition, self-report data may be influenced by participants’ subjective perceptions and response tendencies. Future research could combine quantitative measures with qualitative methods, such as interviews, think-aloud protocols, classroom observations, or learning process data. Such mixed-method approaches would allow for triangulation of the findings and provide a deeper understanding of how pre-service teachers use and respond to GenAI in instructional design contexts. A further limitation concerns the use of questionnaire measures in the specific experimental context of GenAI use. In the present study, critical thinking was assessed using a general critical thinking scale. While this scale was appropriate for measuring participants’ general critical thinking dispositions or abilities, it may have been less able to capture the development of critical thinking specifically related to GenAI use. In GenAI-supported learning environments, critical thinking may involve more context-specific evaluative processes, such as verifying the source and accuracy of AI-generated information, recognizing the limitations and potential biases of AI systems, and reflecting on the broader implications of relying on AI-generated outputs. These AI-specific processes may not be fully captured by a general critical thinking measure. Therefore, future research could adopt more targeted measures developed for GenAI use contexts, such as the Critical Thinking in AI Use Scale developed by Lau et al. (2026), to better examine learners’ critical evaluation and reflective engagement when interacting with GenAI-generated information.

Taken together, these limitations indicate that the findings should be interpreted with caution. Rather than providing conclusive evidence regarding the effects of unscaffolded GenAI use, this study offers exploratory insights into how pre-service teachers may engage with GenAI in instructional design tasks when explicit scaffolding is absent. These insights may inform future research on the design of more targeted and pedagogically grounded scaffolds for GenAI-supported instructional design.

## Figures and Tables

**Table 1:** Pre-test equivalence between the experimental and control groups.

Variable	Group	M	SD	t	*p*
Instructional design Performance	EG	61.77	9.14	−0.92	0.362
	CG	64.00	8.32		
AI Readiness	EG	3.75	0.37	−0.54	0.594
	CG	3.81	0.41		
Self-Regulated learning	EG	4.07	0.47	0.18	0.855
	CG	4.04	0.39		
Critical Thinking	EG	3.97	0.43	−0.98	0.334
	CG	4.09	0.47		

**Table 2:** Results of the ANCOVA on instructional design performance.

Group	N	M	SD	Adj. M	SE	F	*p*	*η* ^2^
CG	26	86.90	6.67	86.54	1.14	8.348	0.006	0.146
EG	26	81.50	6.11	81.87	1.14			

Adj. M = adjusted mean; SE = standard error.

**Table 3:** Results of the ANCOVA on AI readiness.

Group	N	M	SD	Adj. M	SE	F	*p*	*η* ^2^
CG	26	4.11	0.37	4.11	0.07	0.198	0.658	0.004
EG	26	4.07	0.37	4.07	0.07			

Adj. M = adjusted mean; SE = standard error.

**Table 4:** Results of the ANCOVA on SRL.

Group	N	M	SD	Adj. M	SE	F	*p*	*η* ^2^
CG	26	4.15	0.43	4.15	0.08	0.001	0.977	0.000
EG	26	4.15	0.42	4.15	0.08			

Adj. M = adjusted mean; SE = standard error.

**Table 5:** Results of the ANCOVA on critical thinking.

Group	N	M	SD	Adj. M	SE	F	*p*	*η* ^2^
CG	26	4.05	0.42	4.05	0.08	0.000	0.997	0.000
EG	26	4.04	0.43	4.05	0.08			

Adj. M = adjusted mean; SE = standard error.

**Table 6:** Results of paired-samples t-test in the experimental group.

Variable	Group	M	SD	t	*p*
Instructional design performance	Pre	61.769	9.139	−11.443	<0.001
	Post	81.500	6.114		
AI Readiness	Pre	3.748	0.368	−3.274	0.003
	Post	4.066	0.373		
Self-Regulated learning	Pre	4.066	0.469	−0.736	0.468
	Post	4.154	0.421		
Critical thinking	Pre	3.968	0.427	−0.640	0.528
	Post	4.038	0.428		

**Table 7:** Results of paired-samples t test in the control group.

Variable	Group	M	SD	t	*p*
Instructional design performance	Pre	64.000	8.319	−15.415	<0.001
	Post	86.904	6.674		
AI Readiness	Pre	3.806	0.409	−2.769	0.010
	Post	4.114	0.372		
Self-Regulated learning	Pre	4.044	0.387	−0.978	0.338
	Post	4.148	0.433		
Critical thinking	Pre	4.090	0.472	0.326	0.747
	Post	4.051	0.424		

## Data Availability

The data supporting the findings of this study are not publicly available as unrestricted open data but may be shared for academic research purposes upon reasonable request to the corresponding author.

## Abbreviations

GenAI	generative artificial intelligence
SRL	self-regulated learning
CG	control group
EG	experimental group
ANCOVA	analysis of covariance
M	mean
SD	standard deviation
SE	standard error
